# Authorized Shared Electronic Medical Record System with Proxy Re-Encryption and Blockchain Technology

**DOI:** 10.3390/s21227765

**Published:** 2021-11-22

**Authors:** Weizhe Chen, Shunzhi Zhu, Jianmin Li, Jiaxin Wu, Chin-Ling Chen, Yong-Yuan Deng

**Affiliations:** 1 School of Computer and Information Engineering, Xiamen University of Technology, Xiamen 361024, China; wzchen@stu.xmut.edu.cn (W.C.); lijm@xmut.edu.cn (J.L.); wujiaxin1996@stu.xmut.edu.cn (J.W.); 2 School of Information Engineering, Changchun Sci-Tech University, Changchun 130600, China; 3 Department of Computer Science and Information Engineering, Chaoyang University of Technology, Taichung 41349, Taiwan; allendeng@cyut.edu.tw

**Keywords:** EMR sharing, consortium blockchain, proxy re-encryption, privacy protection, IoT, BCoT

## Abstract

With the popularity of the internet 5G network, the network constructions of hospitals have also rapidly developed. Operations management in the healthcare system is becoming paperless, for example, via a shared electronic medical record (EMR) system. A shared electronic medical record system plays an important role in reducing diagnosis costs and improving diagnostic accuracy. In the traditional electronic medical record system, centralized database storage is typically used. Once there is a problem with the data storage, it could cause data privacy disclosure and security risks. Blockchain is tamper-proof and data traceable. It can ensure the security and correctness of data. Proxy re-encryption technology can ensure the safe sharing and transmission of relatively sensitive data. Based on the above situation, we propose an electronic medical record system based on consortium blockchain and proxy re-encryption to solve the problem of EMR security sharing. Electronic equipment in this process is connected to the blockchain network, and the security of data access is ensured through the automatic execution of blockchain chaincodes; the attribute-based access control method ensures fine-grained access to the data and improves the system security. Compared with the existing electronic medical records based on cloud storage, the system not only realizes the sharing of electronic medical records, but it also has advantages in privacy protection, access control, data security, etc.

## 1. Introduction

### 1.1. Background

E-health appeared in the early 21st century; it refers to the use of modern information and communication technology, to provide medical services in the health sector [1]. Electronic medical records (EMRs) is a hot topic in the field of e-health [2]. According to one survey [3], as of March 2017, 86% of provincial hospitals and 75.6% of municipal hospitals have established EMR databases; only 32% of provincial hospitals and 35.2% of municipal hospitals have established electronic medical record information platforms. Although the goal of establishing electronic medical record systems has been achieved throughout the world, there is not enough medical record data sharing.

Electronic medical records (EMRs) involve systematic collections of patient- and population-based health data, which are stored electronically in digital format [4,5]. Effective EMR implementation and networking can save more than USD 81 billion annually, by improving medical efficiency and security [6].

At present, there are problems with the use of EMRs [7,8]. Firstly, data stored in the centralized system databases face many risks, such as hacker intrusions, data stealing, and artificial tampering of data that will endanger data security. Secondly, thousands of medical record data are saved by each regional hospital, separately, and unsystematic storage modes (e.g., the isolation of medical information systems) could result in the database information not being “connected”. Without a unified system for the integration of resources, the sharing of medical data assets cannot be brought into full play, and the efficiency of the entire medical diagnosis is greatly reduced.

In a traditional EMR management system [9,10], the data are invisible, unmanageable, and uncontrollable for patients. Patients do not know which electronic medical record data are stored at a hospital, whether the electronic medical records stored in the cloud are used by a hospital, or shared by other hospitals, or whether the data are leaked. Moreover, even when electronic medical record data are leaked, they cannot be traced.

In January 2009, Satoshi Nakamoto invented bitcoin [11] and proposed a blockchain technology; then, Ethereum [12] expanded the concept of a smart contract, a consensus mechanism [13], and a point-to-point network [14]. Blockchain technology has the characteristics of peer-to-peer computing that is open and transparent, as well as communication security [15] that is difficult to tamper with and has multi-party consensus. It proposed a new solution to the disadvantages of the centralized network, and provided a new feasible paradigm for sharing electronic medical records [16]. Blaze et al. designed a proxy re-encryption scheme [17] in 1998, for the first time, to provide access control to data, to share encrypted data in a third-party server. These technologies bring new hope on how to achieve secure access control for EMR sharing.

### 1.2. EMR Sharing Advantages, threat Models, and Knowing Attacks

The EMR sharing system has the following advantages [18]:

1. The electronic medical record sharing system integrates electronic medical record data between hospitals so that it can be used and browsed across different hospitals, ensuring the availability and accuracy of electronic medical record data.

2. The establishment of a reasonable and effective electronic medical record sharing system facilitates a doctor’s access to a patient’s medical history, significantly reducing the costs associated with a patient’s repeat examinations, and improving the efficiency of treatment.

3. Patients benefit from the sharing of electronic medical records because they can directly query their medical records, examination reports, and drug use in the hospital on the relevant networks.

4. The sharing of electronic medical records contributes to public health safety. The sharing of electronic medical records aids in the monitoring of the epidemic situation, allowing for early prevention and treatment of the epidemic situation, preventing the spread of large-scale infection, and preventing the occurrence of public health emergencies.

However, we may come across some potential threats and attacks while using the system. To make the proposed scheme more effective and safe, we analyze potential threats and attacks. The threat models and knowing attacks are as follows:

1. Data integrity issue [19].

In an insecure network environment, any information transmitted is vulnerable to tampering attacks, so that the data received by the receiver is not the original data. Data integrity is threatened. Therefore, it is necessary to ensure the integrity of the transmission data and protect it from tampering during transportation.

2. Illegal access issue [20].

Unauthorized access refers to the unauthorized use of network resources or the use of network resources in an unauthorized way. In this scheme, users are not allowed to operate other people’s data in an unauthorized way.

3. Forgery and tampering [21].

If an attacker forges or tampers with the data stored in the shared electronic medical record system, it will have a significant impact on the entire system, resulting in massive data loss and errors. As a result, leveraging the non-tampering ability of the blockchain could significantly improve data security.

4. Replay Attack [22].

Replay attack means that the same information or data are repeatedly sent twice or more. If the receiver does not take relevant measures and continuously receives information, it would not be able to effectively identify that the data were received, which would lead to replay vulnerabilities.

5. Collusion Attack [23].

In the proxy re-encryption scheme, if the proxy colludes with the authorized party, the data and encryption key of the authorizer may be decoded. Therefore, in a proxy re-encryption scheme, we must verify whether the scheme can resist collision attacks.

Based on the above situation, this study proposes an EMR data sharing mechanism based on the advantages of anti-tampering and traceability of the consortium blockchain and the security authorization characteristics of proxy re-encryption. The proposed scheme, such that hospitals join the medical consortium blockchain, store the EMR data generated by patients in the consortium blockchain service center, and protect the data security according to the relevant national laws and regulations. At the same time, the chaincode functions are used to realize the EMR, and the proxy re-encryption is used for sharing and authorization. Furthermore, we write the attributes of users and devices into the digital certificate to provide different data access functions for users and devices with different attributes.

The rest of this paper is organized as follows. In Section 2, we review some preliminaries. The system model and detailed design are introduced in Section 3 and Section 4, respectively. The analysis of the system is given in Section 5. Finally, Section 6 concludes the paper.

## 2. Preliminary

### 2.1. Blockchain and Smart Contract (Chaincode)

Blockchain is a kind of chain data structure that combines data blocks with time sequences [24]. A smart contract was a concept proposed by Nick Szabo in the 1990s [25], which is almost the same age as the internet. He defines a smart contract as a set of commitments defined in digital form, including agreements in which contract participants can execute these commitments automatically [26]. In this article, we refer to smart contracts as chaincodes, which are programs that are deployed and run on the blockchain network. The chaincode presets some conditions and rules to trigger the execution of the chaincode under certain events and conditions. The goal of the chaincode is to generate ledger data on the blockchain, which means that all operations on the blockchain data are completed by the chaincode. Moreover, security policies (including data encryption and decryption, data signature and signature verification, access control) will be automatically invoked through chaincodes.

### 2.2. Blockchain of Things 

In ordinary internet of things devices [27], there are problems, such as poor data privacy and difficulty accessing data safely. The blockchain will have a significant impact on the internet of things due to its peer-to-peer, open and transparent communication, secure communication, difficulty to tamper with, and multi-party consensus. The encryption mechanism and data storage characteristics of blockchain just meet the security requirements of the internet of things. The integration of blockchain and the internet of things is called the blockchain of things (BCoT) in academia.

### 2.3. QR Code

A QR code [28], a kind of readable bar code, can identify the binary data recorded in it and obtain the information contained in it by scanning the QR code. A QR code’s characteristics includes the following: it has large information capacity, high decoding reliability, and adopts certain security encryption measures. A QR code is widely used in near-field secure data exchange.

### 2.4. Elliptic Curve Digital Signature Algorithm (ECDSA)

In an elliptic curve system, we need to use a much shorter key than RSA to achieve the same security strength [29]. The elliptic curve digital signature algorithm (ECDSA) is the elliptic curve analog of the digital signature algorithm [30].

We assume that the sender sends *M* and signs *M* to a receiver, and the receiver needs to verify the signature to ensure the correctness of *M*. We assume that the sender has already generated their own key-pairs, G(x,y) is the base point on the elliptic curve $E/F_{n}$ (based on P256 curve), and satisfies *nG* = 0, *n* is a big prime number and is also the order of *G*.

Select a random number $Sk_{A}\in [1,n-1]$ as a private key, and a public key is $Pk_{A}=Sk_{A}G$.

Suppose the sender’s key pair is $\left(Sk_{A},Pk_{A}\right)$.

#### 2.4.1. Signature Generation

(1) Select a random number r∈[1,n−1].
(2) Calculate $R=rG=(x_{1},y_{1})$.
(3) Calculate $S=(SHA(M)+Sk_{A}x_{1})\cdot r^{-1}$ according to random number *r*, private key $Sk_{A}$, and SHA(M), which is the secure hash value of message *M*.
(4) Send message *M*, and signature (R,S) to the receiver.

#### 2.4.2. Signature Verification

(1) Receiver receives *M* and signature (R,S).
(2) Hash SHA(M) according to message *M*.
(3) Use the sender’s public key $Pk_{A}$ to calculate $SHA(M)G/S+x_{1}Pk_{A}/S$ and compare it, if it is equal to *R*:
  $$\begin{array}{l} \;SHA(M)G/S+x_{1}Pk_{A}/S\\ =SHA(M)G/S+x_{1}Sk_{A}G/S\\ =(SHA(M)+x_{1}Sk_{A})G/((SHA(M)+Sk_{A}x_{1})\cdot r^{-1})\\ =rG\\ =R \end{array}$$

If it is equal, the signature verification is successful.

For reading convenience, in the following sections, we use $Sig_{SkX}(\cdot )$ to represent **the signature generation** and $Sign_{X}$ to represent the signature (R,S); we use $Ver_{PkX}(\cdot )$ to represent **the signature verification**.

### 2.5. The Algorithm Elliptic Curve Based Proxy Re-Encryption

In elliptic curve based proxy re-encryption [31], let *E* be an elliptic curve over a limited field *F_q_*, where *q* is a large prime number, and *G* is a point on the elliptic curve *E* of order *n*. Let $G_{1},G_{2}$ be two multiplicative cyclic groups of prime modulo *n*. Let $e:G_{1}\times G_{1}\to G_{2}$ be a bilinear map [32], *z* = $e(G_{1},G_{1})\in G_{2}$.

**Key-Generation:** let private key $a\in Z_{n}^{*}$ and a public key $Pk_{a}=aG\in$ point on *E*.

**Encryption:** generate an arbitrary number $r\in Z_{n}^{*}$, and output $C_{a}=(A,B)=(r\cdot Pk_{a},z^{r}G+P_{m})$, where $P_{m}=f(m)$ [33].

**Re-Key-Generation:** the re-encryption key $rk_{a\to b}=a^{-1}bG$ is generated by private key *a* and public key bG.

**Re-Encryption**: given $rk_{a\to b}$ and message $C_{a}$ computes $C_{b}=(A^{\prime },B^{\prime })=\left(e(raG,a^{-1}bG),z^{r}G+P_{m}\right)$ = $\left(z^{rb},z^{r}G+P_{m}\right)$.

**Decryption:** (1) given ciphertext $C_{a}$ and private key *a*, output $P_{m}=B-(e(A,Sk_{a}^{-1}G))G$; (2) given $C_{b}$ and private key $Sk_{b}$, compute $P_{m}=B^{\prime }-{\left(A^{\prime }\right)}^{1/b}G$; (3) compute *m* = $f^{-1}(P_{m})$.

For reading convenience, in the next section, we use $Enc_{PkX}(\cdot )$ for **Encryption**, reKeyGen(·) for **Re-Key-Generation**, $reEnc_{A\to B}(\cdot )$ for **Re-Encryption**, and $Dec_{SkX}(\cdot )$ for **Decryption**.

## 3. The System Model

### 3.1. The Application Scenario

**Figure 1:**
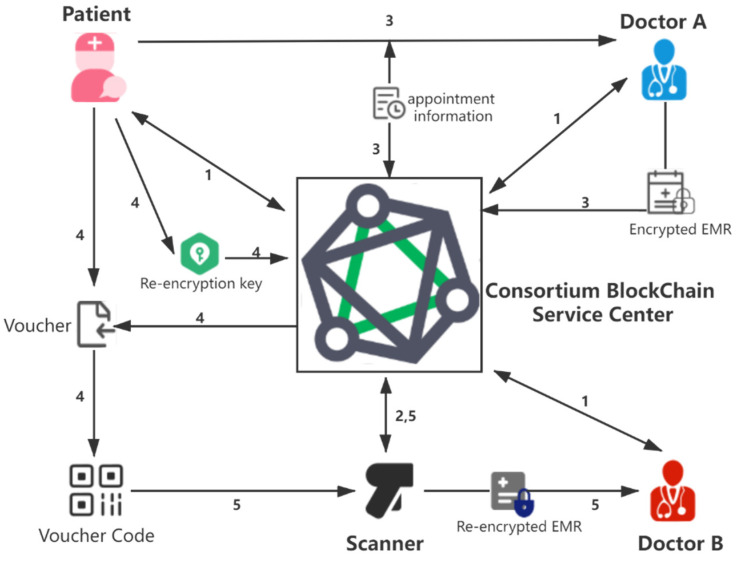
The System Framework.

Figure 1 is the application scenario of the proposed scheme.

In the proposed framework, there are four roles in the scheme.

(1) Consortium blockchain service center (CBSC): the distributed cloud storage and the peer-to-peer network of each hospital organization node constitute the consortium blockchain service center. In fact, the consortium blockchain service center is a decentralized network service. For the convenience of understanding, we call it CBSC. The consortium blockchain service center implements identity management and digital certificate issuance, and chaincode operation, data storage, and data legitimacy verification.
(2) Patient (P): the patient can make an appointment with a doctor. The patient uses the voucher and the scanner to authorize the doctor to view the patient’s historical medical records. The patient authorizes the doctor to view his/her historical medical record through the combination of vouchers generated by proxy re-encryption and scanner.
(3) Doctor (D): the doctor obtains the patient’s appointment information through the CBSC and generates the EMR with the encrypted fields by the patient’s public key after diagnosis and stores it in CBSC.
(4) Scanner (SC): the scanner is used to scan the voucher code presented by the patient and request CBSC to obtain the re-encrypted ciphertext data through the voucher, which CBSC will convert the ciphertext through the re-encryption key.

1. **User registration phase:** all users (including patients and doctors) must register in the CBSC and obtain the private key and the corresponding X.509 digital certificate. The digital certificate contains the user’s role attribute information (doctors, patients) and the user’s public key.
2. **Device registration phase:** the scanner has to register in CBS to obtain the private key and the corresponding X.509 digital certificate. The digital certificate contains the hospital information and public key of the equipment.
3. **Appointment and EMR generation phase:** the patient makes an appointment to see doctor A and chains up the appointment information to CBSC. Then doctor A obtains the appointment information from CBSC and makes a diagnosis. After the diagnosis, doctor A generates and chains up the encrypted EMR to CBSC.
4. **The generation of re-encryption key and access voucher phase:** the patient authorizes doctor B to view his/her historical EMR through proxy re-encryption and blockchain-network scanners. Firstly, the patient calculates the re-encryption key through his/her private key and doctor B’s public key and submits it to CBSC. CBSC returns an authorization voucher to the patient. Then, the patient displays the voucher to doctor B in the form of a QR code.
5. **Scanning of access voucher and acquisition of re-encrypted EMR phase:** the scanner scans the QR code to obtain the voucher, and requests the re-encrypted EMR through the voucher from CBSC, which uses the re-encryption key to convert the historical EMR into the re-encrypted EMR. Finally, doctor B obtains the re-encrypted EMR through the scanner and decrypts it through the private key.

### 3.2. The Consortium Blockchain Service Center Architecture

**Figure 2:**
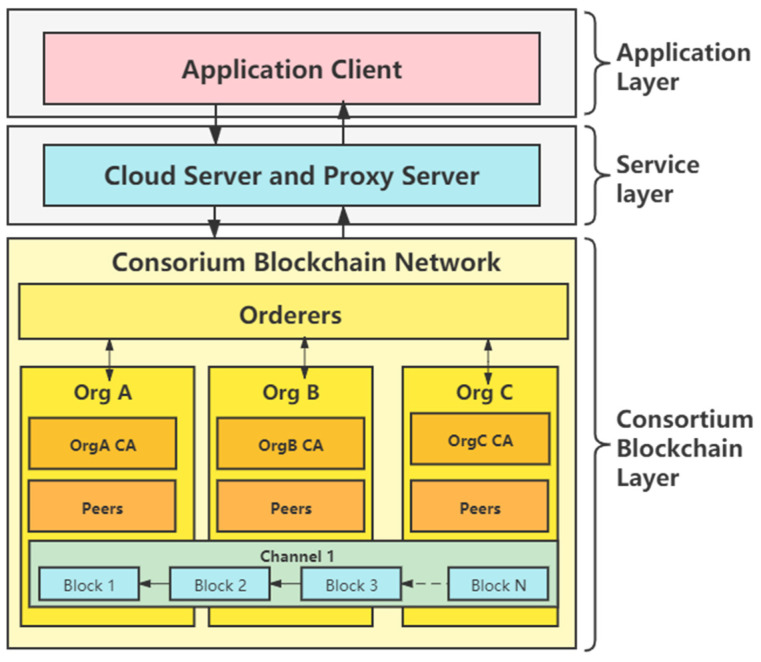
The consortium blockchain service center architecture.

CBSC refers to embedding the blockchain framework into the cloud-computing platform, making use of the deployment and management advantages of cloud service infrastructure to provide users with a convenient and high-performance blockchain ecological environment and ecological supporting services. In this paper, our CBSC architecture is shown in Figure 2, which is divided into the application layer, service layer, and consortium blockchain layer.

**Application layer and service layer:** the application layer provides the EMR function service for patients, doctors, and scanners. It can interact with the background blockchain network via the API provided by the HTTPS server from the service layer. The service layer plays the role of middleware. Besides receiving and processing HTTPS requests from applications, the service layer must interact with the consortium blockchain layer directly to achieve the business logic by invoking a specific chaincode. In this way, the service layer can decouple applications and the data layer.

**Consortium blockchain Layer:** the consortium blockchain layer takes the Hyperledger Fabric technology as the core to provide blockchain services to users. Hyperledger Fabric technology is a new distributed infrastructure and computing mode, which uses chained data structure to verify and store data, uses distributed node consensus algorithm to generate and update data, uses cryptography to ensure the security of data transmission and access, and uses chaincode composed of automatic script code to program and operate data. In fabric, the order nodes use the consensus algorithm to sort the data and packages into blocks. Organizations that join the alliance chain verify and store data through peers.

## 4. The Proposed Scheme

### 4.1. X.509 Digital Certificate

**Figure 3:**
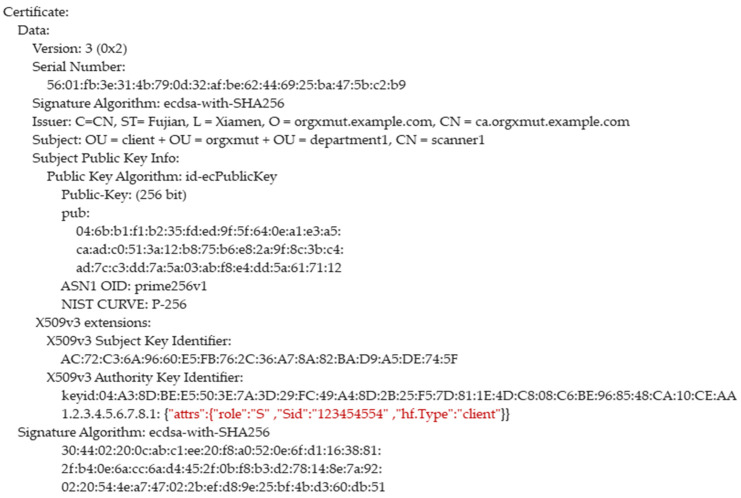
The example of the scanner device digital certificate.

**Figure 4:**
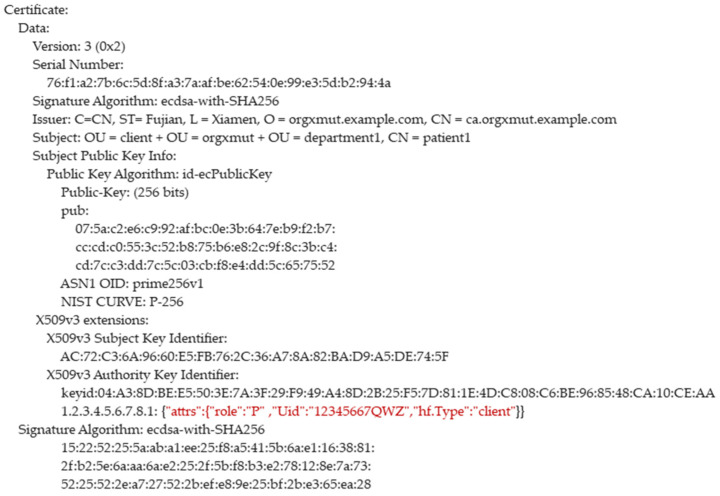
The example of a user’s digital certificate.

CBSC uses X.509 digital certificate to identify the identity of users and devices in CBSC. The most basic information of the X.509 digital certificate includes the public key, the owner information of the public key, and the digital signature of CBSC. The “role” attributes in the digital certificate indicates the registered user or device attribute. If the value of “role” is “D”, it is the doctor user certificate; if the value of “role” is “P”, it is the patient certificate; if the “role” value is “S”, it is the scanner device certificate. The scanner certificate is uniquely identified by the “Sid” attribute of the scanner; in the user certificate, the “Uid” attribute is the user’s identification number and is used as the unique identifier of the use. For the patient, ”Uid” means “Pid”; for the doctor, “Uid” means “Did”. Patients can use the public key in the doctor’s certificate combined with their own private key to realize the proxy re-encryption of data and then realize the access control of data. The examples of the digital certificate are as Figure 3 and Figure 4 shown below.

### 4.2. Deployment and Initialization of the Chaincode

**Figure 5:**
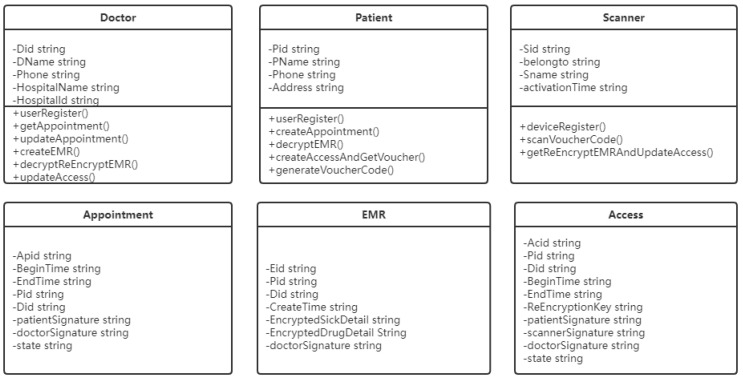
The chaincode data structure.

The chaincode is event-driven, with state storage and programs running on the blockchain. The user realizes data access on CBSC through the chaincode. In this scheme, the chaincode data structure and function of the key information for the proposed architecture we define as Figure 5 follows, and Table 1 shows the detailed introduction of chaincode data structure. In particular, the field “state” of the appointment is divided into two states, namely “CREATE” and “FINISH”. When an appointment is created, the patient needs to sign it and set the state to “CREATE”. After the doctor’s diagnosis, he/she needs to sign and set the state to “FINISH”. In EMR, “EncryptedSickDetail” and “EncryptedDrugDetail” are encrypted fields. When authorizing EMR by using proxy re-encryption, these two fields are re-encrypted. We define the access data structure for access control, in which the “ReEncryptionkey” field is used to re-encrypt the EMR. Similarly, the “state” of access has three states. When the patient creates an access, the state is “CREATE”. When the scanner scans the voucher and obtains the re-encrypted EMR, the state is “SCAN”. When the doctor obtains the EMR, the state is “FINISH”.

### 4.3. Notation

Table 2 shows the notations and their meaning.

### 4.4. User Registration Phase

**Figure 6:**
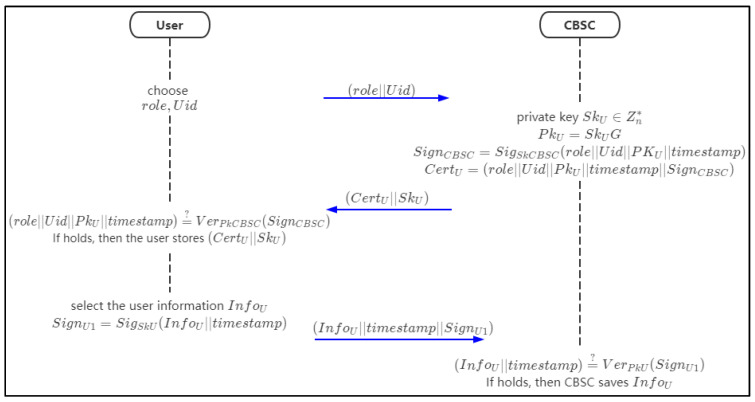
The flow chart of user registration.

Users (including patients and doctors) should register with CBSC. CBSC will generate a private key and digital certificate (including public key) for users, sign the private key and digital certificate, and issue them to users. After the user obtains the private key and digital certificate, the user will verify the correctness of the signature. After the signature is verified successfully, the user will call the chaincode to store the user details in CBSC. The flow chart of the user registration phase is shown in Figure 6.

- Step 1: the user chooses the attributes role and Uid to request for registration in CBSC.
- Step 2: when the CBSC receives the request, it selects a random number as the private key $Sk_{U}$ and uses it with the generator for the elliptic group *G* to compute a public key $Pk_{U}=Sk_{U}G$.

Then the CBSC signs the signature $Sign_{CBS}$ as follows:

(1) $$Sign_{CBSC}=Sig_{SkCBSC}(role||Uid||Pk_{U}||timestamp)$$

- Step 3: CBSC generates digital certificates $Cert_{U}$ for users and sends $Sk_{U}$ and $Cert_{U}$ to users.
  (2) $$Cert_{U}=(role||Uid||Pk_{U}||timestamp||Sign_{CBSC})$$
- Step 4: after receiving the data, the user will verify the correctness of the signature. If it is correct, the certificate is legal.
  (3) $$\left(role||Uid||Pk_{U}||timestamp)\underline{\underline{?}}\;Ver_{PkCBSC}(Sign_{CBSC}\right)$$
  and the user will store the private key $Sk_{U}$ and certificate $Cert_{U}$.
- Step 5: the user selects the user information $Info_{U}$. For a patient, $Info_{U}$ means (PName||Phone||Pid||Address||Phone||CreateTime), as shown in the above patient data structure; for doctors, $Info_{U}=(DName||Phone$||DId||HospitalName||HospitalId||CreateTime) as shown in the above doctor data structure. Then the user signs $\left(Info_{U}||timestamp\right)$
  (4) $$Sign_{U1}=Sig_{SkU}(Info_{U}||timestamp)$$
  and chain up $\left(Info_{U}||timestamp||Sign_{U1}\right)$ to the CBSC.
- Step 6: when the CBSC receives the data from the user, it will verify the signature $Sign_{U1}$
  (5) $$\left(Info_{U}||timestamp)\underline{\underline{?}}\;Ver_{PkU}(Sign_{U1}\right)$$

If it holds, then the CBSC saves the data.

The chaincode of the pseudo-code of user registration is shown in Algorithm 1.
**Algorithm 1.** The chaincode pseudo-code of the user registration information.*user_cert, sk_user = CBSC.CA.CreateIndenity(role,Uid)* *if user_cert != NULL*  *role←user_cert* *if (role == "P")*  *Info←Patient* *else if(role == "D")*  *Info←Doctor* *userSign = ecdsa.sign(sk_user, Info, timestamp)* *UserRegister(Info, timestamp, userSign)*

### 4.5. Device Registration Phase

**Figure 7:**
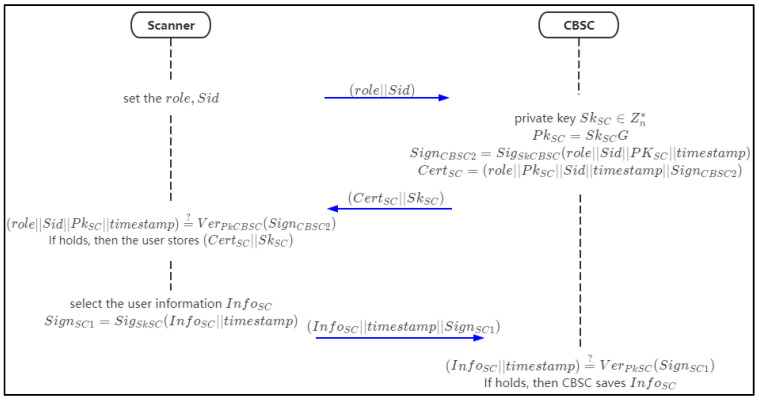
The flow chart of scanner device registration.

The scanner is registered in CBSC under the setting of person. Scanner devices are also registered in CBSC. CBSC will generate a private key and device digital certificate for the scanner, sign the private key and digital certificate, and issue them to devices. After the scanner obtains the private key and digital certificate, it will verify the correctness of the signature. After the signature is verified successfully, the scanner will call the chaincode automatically to store the device details in CBSC. The flow chart of the device registration phase is shown in Figure 7.

- Step 1: the scanner sets the attributes role and *Sid* to request for registration in CBSC.
- Step 2: when the CBSC receives the request, it selects a random number as the private key $Sk_{SC}$ and uses it with the generator for the elliptic group *G* to compute a public key $Pk_{SC}=Sk_{SC}G$ for the scanner.

Then the CBSC signs the signature $Sign_{CBSC2}$ as follows:

(6) $$Sign_{CBSC2}=Sig_{SkCBSC}(role||Sid||Pk_{SC}||timestamp)$$

- Step 3: CBSC generates device digital certificates $Cert_{SC}$ and sends $Sk_{SC}$ and $Cert_{SC}$ to users.
  (7) $$Cert_{SC}=(role||Sid||Pk_{SC}||timestamp||Sign_{CBSC2})$$
- Step 4: after receiving the data, the scanner will verify the correctness of the signature. If it is correct, the certificate is legal.
  (8) $$\left(role||Sid||Pk_{SC}||timestamp)\underline{\underline{?}}\;Ver_{PkCBSC}(Sign_{CBSC2}\right)$$
  and the scanner will store the private key $Sk_{SC}$ and certificate $Cert_{SC}$.
- Step 5: the scanner set up the basic information $Info_{SC}$ and signs $Info_{SC}$ with $Sk_{SC}$,
  (9) $$Sign_{SC1}=Sig_{SkSC}(Info_{SC}||timestamp)$$
  and chain up $\left(Info_{SC}||timestamp||Sign_{SC1}\right)$ to the CBSC.
- Step 6: when the CBSC receives the data from the scanner, it will verify the signature $Sign_{SC1}$
  (10) $$\left(Info_{SC}||timestamp)\underline{\underline{?}}\;Ver_{PkSC}(Sign_{SC1}\right)$$

If it holds, then the CBSC saves the data.

The chaincode of the pseudo-code of device registration is shown in Algorithm 2.
**Algorithm 2.** The chaincode pseudo-code of the device registration.*role,sid←Input()* *(device_cert, sk_sc, timestamp,cbsSign) = CBSC.CA.CreateIndenity(role,sid)* *pk_p = device_cert.getPublickey()* *if ecdsa.verify(cbsSign,{role,sid,pk_p,timestamp})*  *scSign = ecdsa.sign(sk_sc, Info, timestamp)*  *deviceRegister(Info, timestamp, scSign)*

### 4.6. Appointment and EMR Generation Phase

**Figure 8:**
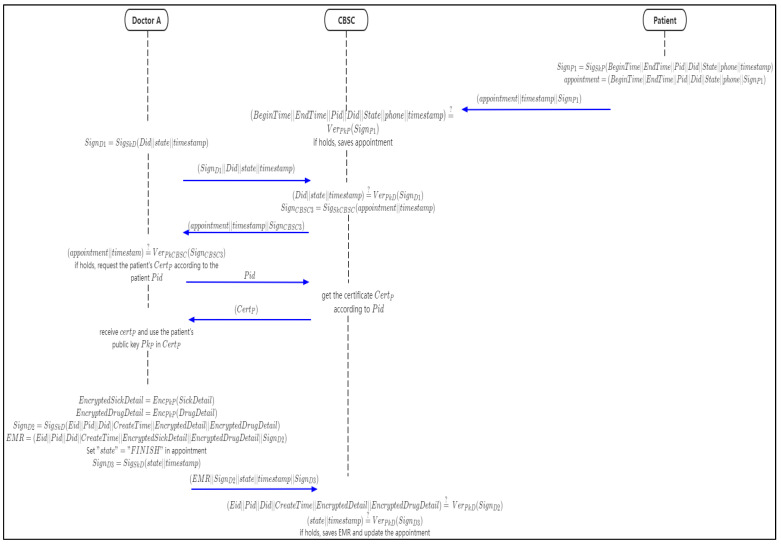
The flow chart of appointment and EMR generation.

In this phase, the patient makes an appointment to see the doctor and chains up the appointment information to the CBSC. Then doctor A obtains the appointment information from CBSC and makes a diagnosis. After diagnosis, doctor A generates and stores an encrypted EMR in CBSC. The flow chart of appointment and EMR generation is shown in Figure 8.

- Step 1: the patient sets the appointment data fields and sets the field “state” = ”CREATE”, then signs them with the patient’s private key $Sk_{P}$,
  (11) $$Sign_{P1}=Sig_{SkP}(BeginTime||EndTime||Pid||Did||state||timestamp)$$
  (12) $$appointment=(BeginTime||EndTime||Pid||Did||state||Sign_{P1})$$

Then sends $\left(appointment||Sign_{P1}||timestamp\right)$ to CBSC.

- Step 2: when CBSC receives the data, it will get the data fields from appointment and verify the data signature,
  (13) $$\left(BeginTime||EndTime||Pid||Did||state||timestamp)\underline{\underline{?}}\;Ver_{PkP}(Sign_{P1}\right)$$

If holds, CBSC will save the data.

- Step 3: Doctor A requests the appointment form; the state field value is “CREATE” in CBSC,
  (14) $$Sign_{D1}=Sig_{SkD}(Did||state||timestamp)$$

Then requests to CBSC with parameters $\left(Sign_{D1}||Did||state||timestamp\right)$

- Step 4: upon receiving the request, CBSC will verify the correctness of the signature,
  (15) $$\left(Did||state||timestamp)\underline{\underline{?}}\;Ver_{PkD}(Sign_{D1}\right)$$

If it holds, then the appointment will be found according to (Did||state), and the appointment will be signed.

(16) $$Sign_{CBSC3}=Sig_{SkCBSC}(appointment||timestamp)$$

and sent to doctor A with $\left(appointment||timestamp||Sign_{CBSC3}\right)$.

- Step 5: after receiving the data, doctor A will verify the correctness of the data,
  (17) $$\left(appointment||timestamp)\underline{\underline{?}}\;Ver_{PkCBSC}(Sign_{CBSC3}\right)$$

If it is held, the patient’s certificate is requested according to the patient’s *Pid*.

- Step 6: when CBSC receives doctor A’s request, it sends the patient’s digital certificate $Cert_{P}$ to doctor A, and doctor A obtains the patient’s public key $Pk_{P}$ from the patient’s digital certificate.
- Step 7: after the diagnosis, doctor A will generate the EMR for the patient and encrypt the EncryptedSickDetail (the encrypted sick detail filed) and EncryptedDrugDetail (the encrypted drug detail filed) fields in the EMR with the patient’s public key.
  (18) $$EncryptedSickDetail=Enc_{PkP}(SickDetail)$$
  (19) $$EncryptedDrugDetail=Enc_{PkP}(DrugDetail)$$

$Sign_{D2}=Sig_{SkD}(Eid||Pid||Did||CreateTime||EncryptedDetail||EncryptedDrugDetail)$

$EMR=(Eid||Pid||Did||CreateTime||EncryptedSickDetail||EncryptedDrugDetail||Sign_{D2})$

Then, set the “state” filed = “FINISH” in appointment and sign it,

(20) $$Sign_{D3}=Sig_{SkD}(state||timestamp)$$

and doctor A sends $\left(EMR||Sign_{D2}||state||timestamp||Sign_{D3}\right)$ to CBSC.

- Step 8: when CBSC receives doctor A’s data, it will verify the correctness of the signature.
  (21) $$\left(Eid||Pid||Did||CreateTime||EncryptedDetail||EncryptedDrugDetail)\underline{\underline{?}}\;Ver_{PkD}(Sign_{D2}\right)$$
  (22) $$\left(state||timestamp)\underline{\underline{?}}\;Ver_{PkD}(Sign_{D3}\right)$$

If the signature is correct, it will store the EMR and update the “state” of the appointment.

Algorithms 3 and 4 show the appointment generation and EMR generation respectively.
**Algorithm 3.** The chaincode pseudo-code of appointment generation.*Did←Doctor.Did* *Pid←Patient.Pid* *BeginTime,EndTime←Input()* *state←“CREATE”* *timestamp←System.currentTime* *if patient_cert != NULL*  *pSign = ecdsa.sign(sk_p,Did,Pid,BeginTime,EndTime,timestamp)*  *Appointment={Apid,BeginTime,EndTime,Pid,Did,pSign,state}*  *createAppointment(Appointment, timestamp, pSign)*

**Algorithm 4.** The chaincode of the pseudo-code of EMR generation.*Did←Doctor.Did* *state←“CREATE”* *timestamp←System.currentTime* *if doctor_cert != NULL*  *dSign1 = ecdsa.sign(sk_d,Did,state,timestamp)*  *(Appointment,timestamp,cbsSign)=getAppointment(Did,state,,timestamp,dSign1)*  *if ecdsa.verify(cbsSign,Appointment,timestamp)*    *Pid←Appointment.Pid*    *patient_cert = CBSC.CA.GetIndentityByAttribute(Pid)*    *pk_p←patient_cert.getPublicKey*    *EncryptedSickDetail = ECC.Encrypt(pk_p, sickdetail)*    *EncryptedDrigDetail = ECC.Encrypt(pk_p, drugdetail)*    *Did←Appointment.Did*    *CreateTime←System.currentTime*    *dSign2 = ecdsa.sign(sk_d,Pid,Did,CreateTime,EncryptedSickDetail,*        *EncryptedDrugDetail)*    *EMR={Eid,Pid,Did,CreateTime,EncryptedSickDetail,EncryptedDrugDetail,dSign2}*    *State←“FINISH”*    *dSign3 = ecdsa.sign(sk_d,state,timestamp)*    *createEMR(EMR)*    *updateAppointment(state,timestamp,dSign3)*

### 4.7. The Generation of Re-Encryption Key and Access Voucher Phase

**Figure 9:**
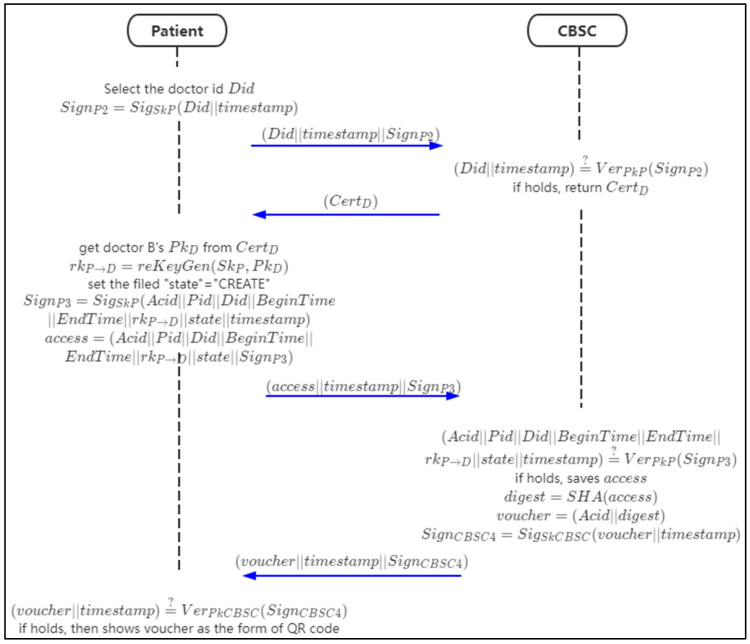
The flow chart of re-encryption key and access voucher generation.

In this phase, the patient will combine his private key with the public key of doctor B to generate the re-encryption key and send it to CBSC. After verification, CBSC will return the access voucher to the patient. The patient displays the access voucher to doctor B in the form of a QR code. Figure 9 shows the data flow in this phase.

Step 1: The patient requests doctor B’s certificate $Cert_{D}$ from CBSC through doctor B’s ID Did.

(23) $$Sign_{P2}=Sig_{SkP}(Did||timestamp)$$

Request with parameters $\left(Did||timestamp||Sign_{P2}\right)$.

Step 2: when CBSC receives the request, it will verify the signature $Sign_{P3}$,

(24) $$\left(Did||timestamp)\underline{\underline{?}}\;Ver_{PkP}(Sign_{P2}\right)$$

If it holds, then return doctor B’s certificate $Cert_{D}$.

Step 3: the patient gets doctor B’s public key $Pk_{D}$ from $Cert_{D}$, then generates the re-encryption key $rk_{P\to D}$.

(25) $$rk_{P\to D}=reKeyGen(Sk_{P,}Pk_{D})$$

Then the patient sets the “state” = ”CREATE” in access, signs the fields of access, and forms access data.

(26) $$Sign_{P3}=Sig_{SkP}(Acid||Pid||Did||BeginTime||EndTime||rk_{P\to D}||state||timestamp)$$

(27) $$access=(Acid||Pid||Did||BeginTime||EndTime||rk_{P\to D}||state||Sign_{P3})$$

Then sends $\left(access||timestamp||Sign_{P3}\right)$ to CBSC.

Step 4: when CBSC receives the data, it will verify the correctness of $Sign_{P3}$ firstly,

(28) $$\left(Acid||Pid||Did||BeginTime||EndTime||rk_{P\to D}||state||timestamp)=Ver_{PkP}(Sign_{P3}\right)$$

If it is held, then save the access and generates the data digest and forms the data voucher,

(29) digest=SHA(access)

(30) voucher=(Acid||digest)

(31) $$Sign_{CBSC4}=Sig_{SkCBSC}(voucher||timestamp)$$

Then sends $\left(voucher||timestamp||Sign_{CBSC4}\right)$ to the patient.

Step 5: when the patient receives the data, the signature will be verified,

(32) $$\left(voucher||timestamp)\underline{\underline{?}}\;Ver_{PkCBSC}(Sign_{CBSC4}\right)$$

If it holds, the voucher QR code will be generated and be shown to doctor B.

Algorithm 5 shows the generation of the registration of the re-encryption key and access voucher.
**Algorithm 5.** The chaincode of the pseudo-code of the generation of re-encryption key and access voucher.*Did←Input()* *Pid←Input()* *dortor_cert←CBSC.CA.GetIndentityByAttribute(Did)* *pk_d←doctor_cert.getPublicKey* *rkey = reKeyGen(sk_p,Pk_d)* *BeginTime,EndTime←Input()* *state = “CREATE”* *timestamp = System.currentTime* *if patient_cert != NULL*  *pSign = ecdsa.sign(sk_p,{Pid,Did,BeginTime,EndTime,rkey,state,timestamp})*  *access = {Acid, Pid,Did,BeginTime,EndTime,rkey,state,pSign}*  *(voucher,timestamp,cbsSign) = createAccessAndGetVoucher(access,timestamp,pSign)*  *If ecdsa.verify(cbsSign,voucher,timestamp)*    *generateVoucherCode(voucher)*

### 4.8. Scanning of Access Voucher and Acquisition of Re-Encrypted EMR Phase

In this phase, the patient authorizes doctor B to view their historical EMR through proxy re-encryption and scanner.

Step 1: doctor B scans the QR code with the scanner and generates the signature

(33) $$Sign_{SC}=Sig_{SkSC}(voucher||timestamp)$$

The scanner requests the data in CBSC $\left(voucher||timestamp||Sign_{SC}\right)$.

Step 2: after receiving the request, CBSC first verifies the correctness of the signature,

(34) $$\left(voucher||timestamp)\underline{\underline{?}}\;Ver_{PkSC}(Sign_{SC}\right)$$

If it holds, CBSC queries the access according to acid, hashes access, and compares it with the digest, $digest\underline{\underline{?}}SHA(access)$. If it is correct, update the *state* field access to “SCAN” and add the scanner’s signature to access.

(35) $$access=(Acid||Pid||Did||BeginTime||EndTime||rk_{P\to D}||state||Sign_{P3}||Sign_{SC})$$

Then CBSC re-encrypts the encrypted field in EMR and sends it to doctor B through the scanner.

(36) $$reEncryptedSickDetail=reEnc_{P\to D}(rk_{P\to D},EncryptedSickDetail)$$

(37) $$reEncryptedDrugDetail=reEnc_{P\to D}(rk_{P\to D},EncryptedDrugDetail)$$

(38) reEMR=(Pid||reEncryptedSickDetail||reEncryptedDrugDetail)

(39) $$Sign_{CBSC5}=Sig_{SkCBSC}(reEMR||timestamp)$$

Then sends $\left(reEMR||timestamp||Sign_{CBSC5}\right)$ to doctor.

Step 3: after receiving the data, doctor B will verify the signature.

(40) $$\left(reEMR||timestamp)\underline{\underline{?}}\;Ver_{PkCBSC}(Sign_{CBSC5}\right)$$

If it is correct, decrypt the data, update the “state” file to “FINISH” in access and signs,

(41) $$SickDetail=Dec_{SkD}(reEncrypyedSickDetail)$$

(42) $$DrugDetail=Dec_{SkD}(reEncryptdDrugDetail)$$

(43) $$Sign_{D4}=Sig_{SkD}(state||timestamp)$$

Request to update “state” field and add doctor B’s signature into access to CBSC.

Step 4: when CBSC receives the data, it will verify the signature $Sign_{D4}$,

(44) $$\left(state||timestamp)\underline{\underline{?}}\;Ver_{PkD}(Sign_{D}\right)$$

if holds, it will update the “state” field of access to “FINISH”, and add doctor B’s signature to access.

(45) $$access=(Acid||Pid||Did||BeginTime||EndTime||rk_{P\to D}||state||Sign_{P3}||Sign_{SC}||Sign_{D4})$$

**Figure 10:**
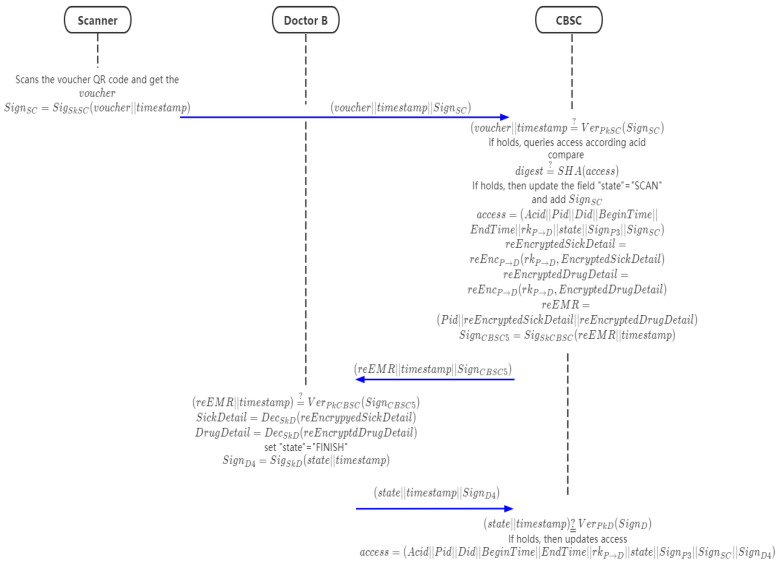
The flow chart of scanning of access voucher and acquisition of re-encrypted EMR.

Figure 10 shows the data flow; Algorithm 6 shows the chaincode in this phase.
**Algorithm 6.** The chaincode of the pseudo-code of scanning of access voucher and acquisition of re-encrypted EMR.*voucher←scanVoucherCode()* *timestamp←System.CurrentTime* *scSign = ecdsa.sign(sk_sc,voucher,timestamp)* *(reEMR,timestamp,cbsSign) = getReEncryptEMRAndUpdateAccess(voucher,timestamp,scSign)* *if ecdsa.verify(cbsSign,reEMR,timestamp)*  *(sickDetail,drugDetail)=decryptReEncryptEMR(reEMR)*  *state = “FINISH”*  *dSign = ecdsa.sign(sk_d,state,timestamp)*  *updateAccess(state,timestamp,dSign)*

## 5. Analysis

### 5.1. Data Integrity Analysis

In order to protect the integrity and security of the data, this paper uses an elliptic curve encryption algorithm (ECDSA) to sign the data.

Taking the user registration phase’s signature as an example, the verification process of the signature $Sign_{CBSC}$ is as follows:

Because $Sign_{CBSC}=(R_{CBSC},S_{CBSC})=(rG,r+hash(role||Uid||Pk_{U}||timestamp||R_{CBSC})Sk_{CBSC})$; therefore, the verification is as follows:

(46) $$\begin{array}{l} E1:R_{CBSC}+hash(role||Uid||Pk_{U}||timestamp||R_{CBSC})Pk_{CBSC}\\ \;\;=rG+hash(role||Uid||Pk_{U}||timestamp||rG)Pk_{CBSC} \end{array}$$

(47) $$\begin{array}{l} E2:S_{CBSC}G=(r+hash(role||Uid||Pk_{U}||timestamp||rP)Sk_{CBSC})G\\ \;\;\;\;\;=rG+hash(role||Uid||Pk_{U}||timestamp||rG)Sk_{CBSC}G\\ \;\;\;\;\;=rG+hash(role||Uid||Pk_{U}||timestamp||rG)Pk_{CBSC} \end{array}$$

When E1 equals E2, the signature verification is correct, which can prove the integrity of the data. Once the data are tampered with, then E1 will not match E2. In this way, the integrity of the data are guaranteed.

**Scene:** the malicious attacker intercepts the information transmitted from CBSC to the user and sends the modified information to the user.

**Analysis:** the attacker will not succeed. The user will verify the integrity of the data:

(48) $$\left(role||Uid||Pk_{U}||timestamp)\underline{\underline{?}}\;Ver_{PkCBSC}(Sign_{CBSC}\right)$$

Because the attacker cannot obtain CBSC’s private key, and if the data are modified, the signature verification will be incorrect, so the attacker will not be able to achieve the purpose of sending the modified data to the user.

### 5.2. Tamper-Resistant

**Figure 11:**
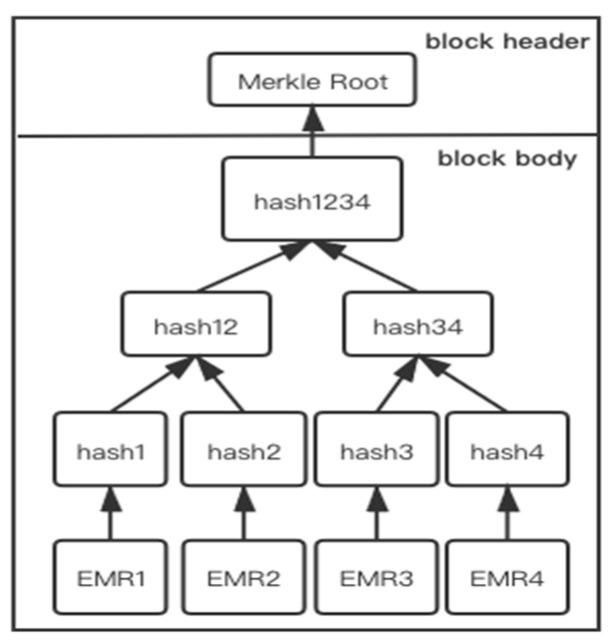
Block structure and Merkle tree in the proposed scheme.

Consortium blockchain technology can ensure that the chain-up information will not be tampered with. All of the chained data stored in a block will be constructed into a binary tree structure of the Merkle tree structure. As shown in Figure 11 below, the hash value between two data records in the Merkle tree will be directly concatenated as the input of the next binary tree. In this way, if an attacker attempts to change any of the data records, the root node of the Merkle tree will change greatly due to the characteristics of the SHA-256 encryption hash, so that other participants will find that the content has been changed when they verify the block information.

### 5.3. Data Security Sharing and Access Control

In the process of authorizing the patient to share the historical EMR with the doctor, the proxy re-encryption algorithm is used to convert the original ciphertext into a ciphertext that can be decrypted by the doctor’s private key. When the doctor wants to view the patient’s historical medical record, the patient will generate the re-encryption key $rk_{P\to D}$ generated by the patient’s private key $sk_{P}$ and the doctor’s public key $Pk_{D}$, and generate access data.

(49) $$access=(Acid||Pid||Did||BeginTime||EndTime||rk_{P\to D}||state||Sign_{P3})$$

Access specifies the usage time (BeginTime and EndTime) of the re-encryption key $rk_{P\to D}$ and uses signature and state to ensure the authenticity and usage record of the data.

Besides, when the users (including patients and doctors) and devices are registered, we write the role attribute into the user or device’s digital certificate (where the patient’s role data are “P”, the doctor’s role attribute is “D”, and the scanner device’s role attribute is “S”). When the user or scanner calls the chaincode to access data, the chaincode will obtain the attribute value in the user or device’s digital certificate firstly, Different chaincode functions and data access are provided according to different attribute values. Table 3 shows the attribute-based access control in this paper.

### 5.4. Blockchain of Things (BCoT)

In this paper, the voucher scanner will be connected to the blockchain. The blockchain-networking scanner will realize the data interaction with the blockchain network through the chaincode (smart contract). Once the chaincode reaches the trigger condition, it will be automatically executed and cannot be tampered with; and the attribute access control is used to specify the chaincode functions that can be accessed by the blockchain-networking scanner. It ensures the device’s secure access to blockchain data.

### 5.5. Known Attacks

#### 5.5.1. Resisting Replay Attack

**Scene:** the information transmitted between sender and receiver might be intercepted by malicious attackers. The attacker mimics the legitimate sender and then sends the same message to the target receiver again.

**Analysis:** because all information transmitted between sender and receiver is protected by ECDSA, and timestamp verification is added, the attacker cannot accurately timestamp parameters, so the attack will fail because the signature verification will fail. Since the information sent after each round will be changed, the same information cannot be sent twice. Therefore, a replay attack cannot succeed in this scheme.

#### 5.5.2. Resisting Collusion Attack

**Scene:** suppose the doctor and the blockchain center (proxy) conspire to obtain the patient’s private key.

**Analysis:** in this scheme, we use the proxy re-encryption scheme, which is collusion resistant. In the phase when the patient authorizes the doctor to view the patient’s historical medical record, doctor B’s public key $Pk_{D}$ is used to calculate the re-encryption key $rk_{P\to D}$ through $Pk_{D}$ and the patient’s private key $Sk_{P}$.

(50) $$rk_{P\to D}=Sk_{P}^{-1}Pk_{D}$$

CBS will convert the encrypted fields in EMR into data that can be decrypted by $Sk_{D}$ through $rk_{P\to D}$. In the whole process, unless the patient exposes his private key, the doctor and the blockchain center (proxy) will not be able to obtain the patient’s private key in collusion.

#### 5.5.3. Man-in-the-Middle Attack

**Scene:** the attacker intercepts the transmitted data and then modifies the intercepted message and sends the modified message to the destination.

**Analysis:** all signatures in the proposed scheme contain a timestamp, and the scheme uses public-key cryptography as well as public and private keys. Therefore, the public key is used to encrypt the data, and the private key is used to sign the data. When the signature involves the private key, the attacker cannot modify the signature or the timestamp. Therefore, they cannot proceed with a man-in-the-middle attack because it is impossible to successfully modify the message.

## 6. Discussion

**Figure 12:**
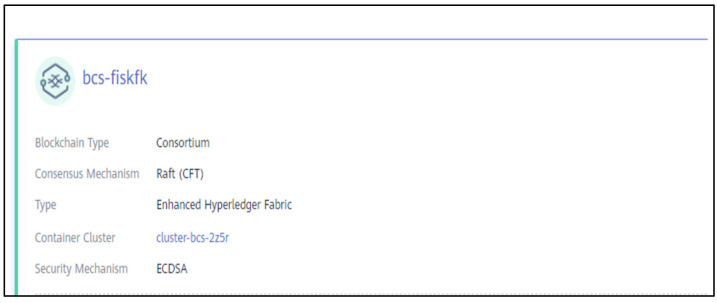
CBSC Configuration.

We test the performance of the blockchain service through the experimental simulation of the mentioned scheme in the following cluster host, as shown in Table 4:

The consortium blockchain service configuration is shown in Figure 12:

### 6.1. Send Rate

**Figure 13:**
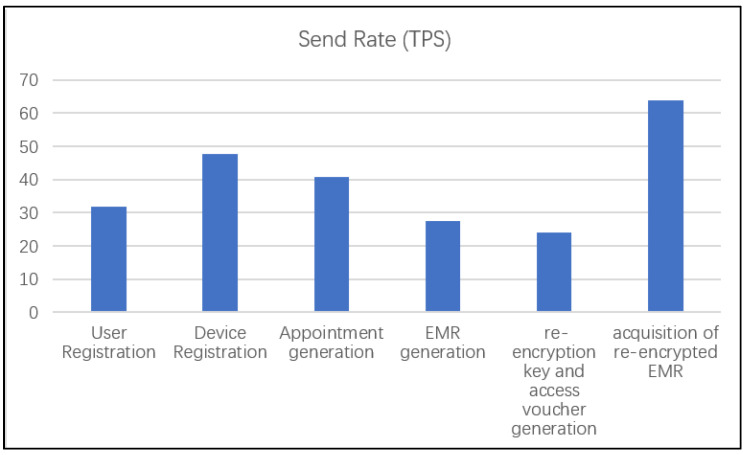
Send rate (TPS).

Caliper is a blockchain performance-testing framework that allows users to test different blockchain solutions using custom use cases, obtaining a set of performance test results. In this scheme, we use the caliper to test the performance of chaincode in five phases, and the results are shown in the figure below. We use 5665 transactions to test, and the sending rate is shown in Figure 13.

### 6.2. System Resource Consumption

The consumption of system resources is as follows. In the simulation experiment of this scheme, we set up two organization nodes, and each organization node consists of a peer node. At the same time, we set the order node, and its system resource consumption is shown in Table 5 below.

### 6.3. The Function Comparison with Other Works

On the subject of patient data confidentiality, Yup et al. [34] investigated the use of blockchain technology in healthcare intelligence. The healthcare data gateway was created to ensure privacy and data access controls were proposed. Liang et al. [35] used blockchain technology to develop a mobile-based healthcare record sharing system, proposing a secure user-centric approach for access control and privacy via a channel formation scheme. Using blockchain, Sun et al. [36] proposed a distributed attribute-based signature scheme for medical systems and a record sharing protocol based on blockchain with supporting algorithms. Using distributed ledger technology, Yang and Li [37] developed an electronic medical record security architecture that improved interoperability between different organizations. The proposed scheme aims to establish a secure electronic medical record sharing system using blockchain smart contracts and cryptography algorithms. Table 6 below compares this work to other related works.

### 6.4. Computation Cost and Communication Cost

#### 6.4.1. Computation Cost

The computation cost of the proposed scheme is shown in Table 7.

#### 6.4.2. Communication Costs

The communication performance of the proposed scheme in the different networks is shown in Table 8.

$L_{Cert}$ is the length of the certificate (5312 bits), $L_{InfoU}$ is the length of $Info_{U}$ (192 bits), $L_{InfoSC}$ is the length of $Info_{SC}$ (128 bits), $L_{Sign}$ is the length of the signature (576 bits), $L_{Sk}$ is the length of the private key (125 bits), $L_{Ap}$ is the length of the appointment data structure (736 bits), $L_{EMR}$ is the length of the electronic medical record data (768 bits), $L_{Ac}$ is the length of access (800 bits), and $L_{Other}$ is the length of other message data (32 bits).

## 7. Conclusions

Blockchain has brought about new ideas to internet medicine. Based on the consortium blockchain technology, this paper implements a sharing EMR system, realizing the following advantages and contributions:

1. The ECDSA signature algorithm and proxy re-encryption algorithm based on ECC were analyzed. Combined with attribute access control, the overall hierarchical architecture of sharing an EMR system based on consortium blockchain with secure access was designed and implemented.

2. According to different role attributes, different chaincodes were designed, and the data access control at the chaincode level was realized through attribute access control.

3. Through the proxy re-encryption algorithm, the data security sharing was realized. The sharing of privacy fields of electronic medical records could be used only with the authorization of patients, which greatly improves the control of patients over their own data.

4. The scanner device was connected to the blockchain network, and the blockchain-networking scanner interacted with the blockchain data through the chaincode, which was executed digitally and automatically. The blockchain-networking scanner used a specific chaincode according to its attributes to realize the device’s secure access to blockchain data.

In future work, we will conduct additional research on the encryption and authorized access of electronic medical records, as well as investigate a more general solution in the form of a security pattern, particularly in fine-grained access to encrypted electronic medical records.

## Figures and Tables

**Table 1:** Data Structure in CBSC.

Data Structure	Meaning
Doctor	The doctor data structure represents the role of the doctor user, where Did is the doctor’s ID, the field DName is the doctor’s name, the *Phone* field refers to the doctor’s mobile phone number, the field HospitalName refers to the name of the doctor’s Hospital, and the HospitalId field refers to the code of the doctor’s hospital. These fields form the basic information of the doctor.
Patient	Patient data structure represents the role of the patient-user, where Pid is the patient’s ID, the field PName is the patient’s name, the field Phone refers to the patient’s mobile phone number, the field Address refers to the name of the patient’s home address.
Scanner	Scanner data structure refers to the basic information of the scanner equipment connected to the network, the Sid field refers to the equipment code, the belongto field refers to the organization, the Sname field refers to the equipment model name, and the field activationTime refers to the activation time of the scanner equipment.
Appointment	Appointment data structure stores the information of the patient’s appointment with the doctor. The BeginTime field refers to the creation time of the appointment, EndTime refers to the end time of the appointment, the Pid field refers to the patient’s ID, Did is the doctor’s ID, PatientSignature refers to the patient’s signature, DoctorSignature refers to the doctor’s signature, the state field refers to the current state of the appointment
EMR	EMR data structure refers to the electronic medical record, which Pid refers to the patient’s ID, Did refers to the doctor’s ID, createTime refers to the creation time of the *EMR*, the EncryptedSickDetail field refers to the encrypted sick detail, the EncryptedDrugDetail field refers to the encrypted drug detail, and doctorSignature refers to the signature of the diagnostic doctor
Access	Access data structure records the information that the patient authorizes the doctor to view the historical EMR. Pid refers to the patient’s ID, Did refers to the doctor’s ID, BeginTime refers to the authorization start time, EndTime refers to the authorization end time, reEncryptionKey refers to the re-encryption key, PatientSignature refers to the patient’s signature, scannerSignature refers to the scanner’s device signature, DoctorSignature refers to the doctor’s signature, and state refers to the current status of access.

**Table 2:** Notations.

Notation	Meaning
G	A generator for the elliptic group
$Pk_{X}$	X’s public key
$Sk_{X}$	X’s private key
role	the *role* is an attribute stored in the digital certificate of the user or device
$Cert_{X}$	X’s X.509 digital certificate
M	X’s original text
$C_{X}$	X’s ciphertext
timestamp	The timestamp of the current time
voucher	Data voucher requesting re-encrypted data
SickDetail	The original detail of sick in EMR
DrugDetail	The original detail of drug in EMR
reEncryptedSickDetail	Re-encrypted sick detail
reEncryptedDrugDetail	Re-encrypted drug detail
reEMR	Re-encrypted EMR
SHA(·)	Secure hash algorithm function
f(m)	The elliptic curve function f(·) of the embedding message m
$P_{m}$	Data embedded in the elliptic curve function f(·)
$f^{-1}(P_{m})$	The inverse function of elliptic curve function f(·), $P_{m}$ is a point on elliptic curve
$info_{X}$	X’s basic information (include name, phone number, and id, etc.)
$rk_{A\to B}$	Proxy re-encryption key generated by *A* and *B*
$A\underline{\underline{?}}\;B$	Determine *A* if equal to *B*
A||B	Concatenate *A* and *B*
$Sign_{X}$	X’s signature
$Sig_{SkX}(M)$	The signature function, use the X’s private key $Sk_{X}$ to sign the message *M*.
$Ver_{PkX}(Sign_{X})$	The verification function, use the X’s public key $Pk_{X}$ to verify the correctness of the signature $Sign_{X}$
$Enc_{PkX}(\cdot )$	The function of encryption with $Pk_{X}$
reKeyGen(·)	The generation of re-encryption keys from *A* to *B*
$reEnc_{A\to B}(\cdot )$	The function of re-encrypting the ciphertext of *A* into the ciphertext that *B* can decrypt.
$Dec_{SkX}(\cdot )$	The function of decryption with the key $Sk_{X}$

**Table 3:** The attribute-based access control in the proposed scheme.

	Function	User Register	Device Register	Appointment Generation	EMR Generation	Re-Encryption Key and Access Voucher Generation	Scanning of Access Voucher and Acquisition of Re-Encrypted EMR
Role							
P		✔		✔		✔	
D		✔			✔		✔
S			✔				✔

**Table 4:** Experimental environment configuration.

Configuration	Detail
CPU	4-core CPU Intel^®^ Xeon^®^ Skylake 6133
Memory	8G
Network	4 Gbit/s
SSD	60 GB

**Table 5:** System resource consumption.

Name	CPU% (max)	CPU% (avg)	Memory (max) (MB)	Memory (avg) (MB)	Traffic In (MB)	Traffic Out (MB)	Disc Write (KB)	Disc Read (KB)
peer0.org1	38.26	18	110	105	11.8	18.2	292	856
peer0.org2	3.19	1.86	54.6	48.5	0.19	0.133	292	68
orderer	1.68	0.26	29.4	27.9	0.1	0.193	288	236

**Table 6:** The function comparison with other works.

Scheme	1	2	3	4	5
Yup et al. [34]	Yes	Yes	Yes	No	No
Liang et al. [35]	Yes	No	Yes	Yes	No
Sun et al. [36]	No	Yes	Yes	No	No
Yang and Li [37]	Yes	Yes	Yes	No	No
proposed scheme	Yes	Yes	Yes	Yes	Yes

1. Architecture. 2. Encryption key. 3. Access control. 4. Authorization sharing. 5. Traceability of access.

**Table 7:** The computation cost of the proposed scheme.

User registration	User	$T_{Cmp}+T_{Sig}$
	CBS	$T_{Sig}+T_{Cmp}$
Device registration	Scanner	$T_{Sig}+T_{Cmp}$
	CBS	$T_{Sig}+T_{Cmp}$
Appointment and EMR generation	Doctor A	$2T_{Enc}+2T_{Sig}+T_{Cmp}$
	CBS	$T_{Sig}+4T_{Cmp}$
	Patient	$T_{Sig}$
The generation of re-encryption key and access voucher	Patient	$2T_{Sig}+T_{Cmp}+T_{RkGen}$
	CBS	$2T_{Cmp}+T_{Sig}+T_{H}$
Scanning of access voucher and acquisition of re-encrypted EMR	Scanner	$T_{Sig}$
	Doctor B	$2T_{Dec}+T_{Sig}$
	CBS	$2T_{RkEnc}+3T_{Cmp}+T_{Sig}$

Notes: $T_{P}$: polynomial function operation; $T_{Cmp}$: comparison operation; $T_{Enc}$: symmetric encryption operation; $T_{Dec}$: symmetric decryption operation; $T_{Sig}$: signature operation; $T_{RkGen}$: re-encrypt key operation; $T_{RkEnc}$: re-encryption operation.

**Table 8:** The communication performance of the proposed scheme in different network.

	Party	Message Length	4G (100 Mps)	5G (20 Gps)
Phase				
1		$L_{Cert}+L_{InfoU}+L_{Sign}+L_{Sk}+3L_{other}$	6301/102,400 ≈ 0.062 ms	6301/20,480,000 ≈ 0.308 us
2		$L_{Sign}+L_{Cert}+L_{InfoSC}+L_{Sk}+3L_{Other}$	6237/102,400 ≈ 0.061 ms	6237/20,480,000 ≈ 0.305 us
3		$5L_{Sign}+2L_{Ap}+L_{Cert}+L_{EMR}+8L_{Other}$	10,688/102,400 ≈ 0.104 ms	10,688/20,480,000 ≈ 0.522 us
4		$3L_{Sign}+L_{Cert}+L_{Ac}+7L_{Other}$	8064/102,400 ≈ 0.079 ms	8064/20,480,000 ≈ 0.394 us
5		$3L_{Sign}+9L_{Other}$	2016/102,400 ≈ 0.02 ms	2016/20,480,000 ≈ 0.098 us

Notes: 1: User registration. 2: Device registration. 3: Appointment and EMR generation. 4: The generation of re-encryption key and access voucher. 5: Scanning of access voucher and acquisition of re-encrypted EMR.

## Data Availability

The data used to support the findings of this study are available from the corresponding author upon request.

## References

[1] Albahri O.S., Albahri A.S., Mohammed K.I., Zaidan A.A., Zaidan B.B., Hashim M., Salman O.H. (2018). Systematic Review of Real-time Remote Health Monitoring System in Triage and Priority-Based Sensor Technology: Taxonomy, Open Challenges, Motivation and Recommendations. J. Med. Syst..

[2] Enaizan O., Zaidan A.A., Alwi N.H.M. (2020). Electronic medical record systems: Decision support examination framework for individual, security and privacy concerns using multi-perspective analysis. Health Technol..

[3] Li C., Xu X., Zhou G., He K., Qi T., Zhang W., Tian F., Zheng Q., Hu J. (2019). Implementation of National Health Informatization in China: Survey About the Status Quo. JMIR Med. Inform..

[4] Mackey T.K., Kuo T.T., Gummadi B., Clauson K.A., Church G., Grishin D., Obbad K., Barkovich R., Palombini M. (2019). ‘Fit-for-purpose?’—Challenges and opportunities for applications of blockchain technology in the future of healthcare. BMC Med..

[5] Halamka J.D., Alterovitz G., Buchanan W.J., Cenaj T., Clauson K.A., Dhillon V., Hudson F.D., Mokhtari M., Porto D.A., Rutschman A. (2019). Top 10 Blockchain Predictions for the (Near) Future of Healthcare. Blockchain Healthc. Today.

[6] Hillestad R., Bigelow J., Bower A., Girosi F., Meili R., Scoville R., Taylor R. (2005). Can electronic medical record systems transform health care? Potential health benefits, savings, and costs. Health Aff..

[7] Sood S.P., Nwabueze S.N., Mbarika V.W., Prakash N., Chatterjee S., Ray P., Mishra S. Electronic medical records: A review comparing the challenges in developed and developing countries. Proceedings of the 41st Annual Hawaii International Conference on System Sciences.

[8] Stafford T.F., Treiblmaier H. (2020). Characteristics of a blockchain ecosystem for secure and sharable electronic medical records. IEEE Trans. Eng. Manag..

[9] Souther E. (2001). Implementation of the electronic medical record: The team approach. Comput. Nurs..

[10] Jung E.Y., Kim J., Chung K.Y., Park D.K. (2014). Mobile healthcare application with EMR interoperability for diabetes patients. Clust. Comput..

[11] Nakamoto S. (2008). A Peer-To-Peer Electronic Cash System. Bitcoin. https://bitcoin.org/bitcoin.

[12] Wood G. (2014). Ethereum: A secure decentralised generalised transaction ledger. Ethereum Proj. Yellow Pap..

[13] Ongaro D., Ousterhout J. In search of an understandable consensus algorithm. Proceedings of the 2014 {USENIX} Annual Technical Conference ({USENIX}{ATC} 14).

[14] Schollmeier R. A definition of peer-to-peer networking for the classification of peer-to-peer architectures and applications. Proceedings of the First International Conference on Peer-to-Peer Computing.

[15] Zyskind G., Nathan O. Decentralizing privacy: Using blockchain to protect personal data. Proceedings of the 2015 IEEE Security and Privacy Workshops.

[16] Azaria A., Ekblaw A., Vieira T., Lippman A. Medrec: Using blockchain for medical data access and permission management. Proceedings of the 2016 2nd International Conference on Open and Big Data (OBD).

[17] Blaze M., Bleumer G., Strauss M. Divertible protocols and atomic proxy cryptography. Proceedings of the International Conference on the Theory and Applications of Cryptographic Techniques.

[18] Zhang X.X., Zhang L. (2012). Construction of Platform for Decision-making Management and Data Center in Hospitals. Chin. Med. Equip. J..

[19] Sattarova Feruza Y., Kim T.H. (2007). IT security review: Privacy, protection, access control, assurance and system security. Int. J. Multimed. Ubiquitous Eng..

[20] Ding S., Cao J., Li C., Fan K., Li H. (2019). A novel attribute-based access control scheme using blockchain for IoT. IEEE Access.

[21] Lee Y., Rathore S., Park J.H., Park J.H. (2020). A blockchain-based smart home gateway architecture for preventing data forgery. Hum.-Cent. Comput. Inf. Sci..

[22] Miao F., Pajic M., Pappas G.J. Stochastic game approach for replay attack detection. Proceedings of the 52nd IEEE Conference on Decision and Control.

[23] Nuñez D., Agudo I., Lopez J. (2017). Proxy re-encryption: Analysis of constructions and its application to secure access delegation. J. Netw. Comput. Appl..

[24] Swan M. (2015). Blockchain: Blueprint for a New Economy.

[25] Szabo N. (1997). Formalizing and securing relationships on public networks. First Monday.

[26] Wang S., Yuan Y., Wang X., Li J., Qin R., Wang F.Y. An overview of smart contract: Architecture, applications, and future trends. Proceedings of the 2018 IEEE Intelligent Vehicles Symposium (IV).

[27] Dai H.N., Zheng Z., Zhang Y. (2019). Blockchain for Internet of Things: A survey. IEEE Internet Things J..

[28] Soon T.J. (2008). QR code. Synth. J..

[29] Johnson D., Menezes A., Vanstone S. (2001). The elliptic curve digital signature algorithm (ECDSA). Int. J. Inf. Secur..

[30] Chandrakar P., Om H. (2017). A secure and robust anonymous three-factor remote user authentication scheme for multi-server environment using ECC. Comput. Commun..

[31] Thangam V., Chandrasekaran K. Elliptic curve based proxy re-encryption. Proceedings of the Second International Conference on Information and Communication Technology for Competitive Strategies.

[32] Zhang F., Safavi-Naini R., Susilo W. An efficient signature scheme from bilinear pairings and its applications. Proceedings of the International Workshop on Public Key Cryptography.

[33] Udin M.N., Abd Halim S., Jayes M.I., Kamarulhaili H. Application of message embedding technique in ElGamal elliptic curve cryptosystem. Proceedings of the 2012 International Conference on Statistics in Science, Business and Engineering (ICSSBE).

[34] Yue X., Wang H., Jin D., Li M., Jiang W. (2016). Healthcare data gateways: Found health care intelligence on blockchain with novel privacy risk control. J. Med. Syst..

[35] Liang X., Zhao J., Shetty S., Liu J., Li D. Integrating blockchain for data sharing and collaboration in mobile healthcare applications. Proceedings of the 2017 IEEE 28th Annual International Symposium on Personal, Indoor, and Mobile Radio Communications (PIMRC).

[36] Sun Y., Zhang R., Wang X., Gao K., Liu L. A decentralizing attribute-based signature for healthcare blockchain. Proceedings of the 2018 27th International Conference on Computer Communication and Networks (ICCCN).

[37] Yang G., Li C. A design of blockchain-based architecture for the security of electronic health record (EHR) systems. Proceedings of the 2018 IEEE International Conference on Cloud Computing Technology and Science (CloudCom).

